# Swedish Child Health Care nurses conceptions of overweight in children: a qualitative study

**DOI:** 10.1186/1471-2296-13-57

**Published:** 2012-06-14

**Authors:** Gabriella E Isma, Ann-Cathrine Bramhagen, Gerd Ahlstrom, Margareta Östman, Anna-Karin Dykes

**Affiliations:** 1 Department of Health Care Sciences, Faculty of Health and Society, Malmö University, Malmö, Sweden; 2 The Swedish Institute for Health Sciences (Vårdalinstitutet), Lund University, Lund, Sweden; 3 Department of Health Sciences, Lund University, Lund, Sweden

**Keywords:** Child, Conceptions, Nurses, Overweight, Perceptions, Primary health care, Qualitative research

## Abstract

**Background:**

Registered Sick Children’s Nurses and District Nurses employed at Child Health Care centres are in a position to help prevent childhood overweight and obesity. Prevention of this challenging public health threat could be improved through having a better understanding of how this group of nurses perceives childhood obesity. The aim of this study was to elucidate the conceptions of childhood overweight, including obesity, among nurses working in Child Health Care.

**Method:**

A qualitative study using a phenomenographic approach, based on open-ended interviews with 18 Child Health Care nurses (CHC-nurses) strategically selected from 17 Child Health Care Centres in the southern part of Sweden.

**Results:**

Four categories of description emerged from the data: Perception of childhood overweight changes, Overweight in younger children a neglected concern, Overweight a delicate issue and Importance of family lifestyle. The participating CHC-nurses conceived overweight in children, primarily obesity in children to be an extensive and serious problem which affects children, families and the surrounding society. Overweight in children was further perceived as a consequence of their parent’s lifestyle and their awareness of the problem, which was considered by the CHC-nurses as a sensitive and a provoking issue. It was also perceived that overweight in children is not taken seriously during the pre-school period and that concerns regarding overweight in younger children were mainly about the appearance and not the health of the child. The CHC-nurses perceived that the proportion of overweight children has increased, which Swedish society and the CHC-nurses have adapted to. This adaptation makes it difficult for CHC-nurses to define those children who are overweight.

**Conclusion:**

CHC-nurses provide a comprehensive and complex picture of childhood overweight, which includes several difficulties dealing with this issue. Attention to CHC-nurse’s conceptions of overweight in children is important since it can affect the parent-nurse relationship and thereby the nurse’s, as well as the parent’s efforts to influence the children’s weight. It is suggested that CHC- nurses should work with person centered counseling and empowerment concerning parent to child relations in cases involving overweight.

## Background

Childhood obesity, has become one of the most challenging public health concerns globally, with an estimated 42 million overweight children under the age of five [1]. It is likely that 25 % of obese children under the age of 6 and 75 % of obese adolescents remain obese into adulthood and thereby develop related diseases such as diabetes and cardiovascular diseases at a younger age [1,2].

Compared to other European countries Sweden has relatively low prevalence of childhood overweight. Currently, about 17 % of Swedish 7-to-9-year-old children are overweight, including 3 % with obesity [3].

There are studies from Sweden that show an escalating increase in the prevalence of childhood overweight over the last three decades [4-6], but recent data indicates that the overweight and obesity rate among children may be leveling off [7,8]. Nevertheless, there are still many overweight children [9] and any country with a high rate of childhood obesity must take action against this extensive health problem and acknowledge that it is the responsibility of all health professionals to work towards the prevention of this health problem [10].

In Sweden, Child Health Care nurses (CHC-nurses), i.e. Registered Sick Children’s nurses (RSCNs) and District nurses (DNs), are those who frequently encounter overweight preschool children and their families through the Child Health Care (CHC). The task of the CHC is to support parents in active parenting and thereby promote the development of children’s health and safety. The role of the nurses is to detect and prevent illness and risks in the child’s proximity from birth to the age of six. The CHC in Sweden is free of charge and should be actively offered to every pre-school child in the country [11]. Since the CHC-nurses have access to almost 100 percent of the children (0–6 year) they have the potential to play an important role in the prevention of childhood overweight and obesity. Hence, the risk of being overweight or obese can be reduced through health promoting activities enhanced by CHC-nurses [12].

However, existing international literature indicates that there are several barriers against the proper management of obesity and one major concern is the attitudes and views of health care personnel [13-16]. Additionally, research has found that obese patients are exposed to negative stereotypes and attitudes held by health care providers. Health care settings are an important source of weight stigma, which undermines these patients opportunity to receive effective medical care [17].

Several qualitative studies by general practitioners (GPs) and nurses working in primary care, have revealed negative beliefs surrounding obesity and obesity care for adult populations [18-22]. Research with focus on the care for overweight children also indicates that there exist negative beliefs among health care workers. A review studying communication issues between health care workers and parents reveal that parents of children who are overweight may be fearful that their child could be stigmatised. Health care personnel’s attitudes and beliefs about obesity can affect their encounter with these families [23] and they often attempt to avoid the topic of overweight in order not to prejudice their relationship with the parents [24]. It is especially difficult for nurses to raise issues related to a child’s excess weight if the parents are overweight themselves [25]. Walker et al, [26] found that GPs and nurses believe that their responsibility is to raise the issue of a child’s weight with its parents but that childhood obesity is a social and family problem in the end to be dealt with outside the scope of public health care. Moreover, barriers like difficulties and inconvenience when counseling children and their parents about weight are also described by school nurses [15].

Learning from existing literature related to perceptions of childhood overweight it is clear that the area of research regarding the perceptions of childhood overweight held by CHC- nurses has not been given much attention. It is also obvious that perceptions of overweight have an impact on the behavior of both health care personnel and parents. Perceptions can be influenced and more important, perceptions can change. Raising awareness of existing perceptions related to children with overweight might prevent these children from the risk of being stigmatized by CHC-nurses and other health care personnel. Furthermore, knowledge of the CHC-nurses perceptions of childhood overweight could assist in the identification of areas that need to be addressed to enable CHC-nurses to be actively involved in the prevention of overweight and obesity among children. Nevertheless, overweight and obesity, as well as their complications, might be preventable. Therefore the prevention of childhood obesity requires urgent attention [1].

Against this background, the aim of the present study was to elucidate conceptions of childhood overweight, including obesity, among CHC-nurses.

## Methods

A phenomenographical approach was chosen for the study, which includes two research perspectives: the first order perspective, which is the description of the world as it is and the second order perspective, which is the description of the world as it is experienced [27]. The second order perspective is emphasised in phenomenography and interviews are the most common way of collecting data. The method was developed in the 1970s by Marton and his co-workers, and originates from an empirical educational framework. The focus is on how people experience and perceive a given phenomenon and not on the phenomenon itself. The interest lies in the variety of ways humans have of understanding the world around them [27].

Individual interviews present different conceptions which form categories of description in an analysis. The categories of description represent a collective level of the various conceptions [27,28].

### Participants

The professional background of the CHC-nurses involves RSCNs who are registered nurses with one year of specialisation in health- and medical care of children and young people, while DNs are registered nurses with 1.5 year of specialisation in primary health- and medical care of children, adults and the elderly. The selection criteria for the participants in this study were; CHC-nurses with at least one year of clinical experience working at one of the 26 CHCs located in four municipalities in the southern part of Sweden and with experience of working with overweight children. In order to ensure a broad range of conceptions, a strategic selection of both CHCs and participants was made. The CHCs (n = 17) were stratified after catchment area and population in order to get a representative sample of the whole population, whilst the CHC-nurses were stratified after age, gender, nationality and professional experience in CHC. Overall, 91 CHC-nurses were eligible for this study. Originally, 19 RSCNs and DNs were invited to participate, however, one nurse declined due to workload. The decision to stop at 19 nurses was taken because no new information was added and these 19 nurses stratified by age, gender, nationality and professional experience in CHC were a representative sample of the 91 eligible nurses and each one of the 17 CHCs. Thus, 18 CHC-nurses were interviewed at 17 CHCs, two of the participants at the same location.

### Procedures and data collection

The local director of the Primary Health Care was personally informed about the study. Written agreement for CHC-nurses to participate was obtained and the responsible manager at each CHC was formally informed about the study by the director of the Primary Health Care.

The presumptive participants were initially approached by e-mail and two weeks later by telephone. The participants received the same written and oral information as their managers. The interviews were made exclusively by the first author (GEI) during 4 months in 2010. The participants were allowed to choose the time and the preferred setting for the interview during their working hours. Each interview began with the participant being offered written and oral information and a request to give their informed consent. All of the interviews took place at the offices of the participants and the interviews were digitally recorded and transcribed verbatim. The interviews were then listened to and read through by the interviewer (GEI) to ensure that they were transcribed correctly. The first interview was also read through and commented by the last author of this study (AKD).

#### Interviews

The interviews were held in conversation form and each interview was supported with an interview guide developed by two of the researchers (GEI, AKD). The open-ended interview guide had one initial question: What is your perception of childhood overweight and obesity? Depending on the character of the answer the responses were penetrated using further questions. It was each informant’s choice of focus that developed the character of the interview. The interview guide included supplementary questions such as; what do you mean? Can you clarify? Can you develop your thoughts? In what way? Can you exemplify? Is there anything you want to add? The interviews lasted between 26 to 90 minutes (median 46 minutes).

### Data analysis

The analysis was carried out in five-stages inspired by the phenomenographic approach [22,28,29]. Stage 1, the transcripts were read through several times by the authors GEI, AKD and ACB in order to gain an overall meaning. Stage 2, the statements relevant to the study were identified according to each of the participant nurse’s different ways of perceiving overweight in children (GEI, AKD). The statements were continuously compared and sorted into different preliminary conceptions and according to the themes addressed (GEI, AKD). Stage 3, the identified conceptions were then compared on the basis of there being any differences and similarities in relation to each other, which would entail some revision. The conceptions were grouped according to their common features into preliminary categories of description. Stage 4, the final categories of description, containing each of the varieties of conception were decided and individually labeled (GEI, AKD, ACB). Stage 5, the focus shifted to the relations between the categories of description. The elements of the phenomenon were identified within each category of description and a description of each conception was summarised (GEI, AKD, ACB, GA, MÖ). Finally, the categories of description was scrutinised to ensure their coherence with the conceptions (GEI, AKD, GA).

### Ethical considerations

Approval for the study was granted by the Regional Ethical Review Board in Lund, Sweden (Dnr. 2010/694). The interviews were voluntary and were conducted according to the Declaration of Helsinki [30].

## Results

The age of the participants in the study group ranged from 32 to 63 years with a mean age of 51 years. The professional background of the CHC-nurses was spread as follows; 10 RSCNs, 5 DNs and 3 CHC-nurse were both RSCNs and DNs. The participant’s years of professional experience amounted to a mean of 11 years, ranging from 1–29 years (Table 1).

**Table 1:** Demographic characteristic of the nurses at Child Health Care (CHC)

Characteristics	**CHC-nurses** (total, n= 18) [%]
**Gender**	
Male	1[6]
Female	17 [94]
**Age**	
30 - 40	3 [17]
41 - 45	2 [11]
46 - 50	4 [22]
51 - 55	2 [11]
56 - 65	7 [39]
**Country of birth**	
Sweden	15 [83]
Other	3 [17]
**Years in profession**	
1 - 5	6 [33]
6 - 10	5 [28]
11 - 16	1 [6]
17 - 20	5 [28]
21 - 25	-
26 - 30	1 [6]

The main findings constituted four categories of description illuminating CHC-nurses conceptions of overweight in children (Table 2). The conceptions in each category of description are described in the current text and are illuminated by selected quotes from interviewees. Each quote is followed by a bracket containing a numerical code, representing one specific interviewee and one interview. For the sake of readability, the concept overweight is further used in the text as a comprehensive term covering both overweight and obesity.

**Table 2:** CHC-nurses’ conceptions of childhood overweight in Child Health Care (CHC)

**Categories of description**	**Conceptions**
**Perception of childhood overweight changes**	*Acceptance of children’s excess weight*
	*Health seems less important than appearance*
**Overweight in younger children a neglected concern**	*Minimisation of the concern for younger children*
	*Emphasis mainly on diet*
**Overweight a delicate issue**	*Overweight a sensitive subject*
	*Overweight in children a provoking concern*
**Importance of family lifestyle**	*Consequences of parents stressed lifestyle*
	*Economic conditions and low education level as contributing factors*
	*Preferences differ in socio-culture*
	*Awareness of the problem*
	*Problem of setting boundaries*

### Perception of childhood overweight changes

This category summarises CHC-nurses conceptions concerning what role the Swedish society plays in the concept of overweight in children.

#### Acceptance of children’s excess weight

CHC-nurses conceived that the views held by Swedish society and health care professionals, related to the healthy weight of pre-school children, have changed in recent years. According to the participant CHC-nurses, the increased proportion of overweight children means that these children no longer stand out. Their conception was that those children who were earlier regarded as having a healthy weight were now regarded as being thin. The Swedish population has become heavier in general and this is something that the CHC-nurses regarded as being due to the fact that the Swedish society has accepted the situation and adapted to it. Moreover, the participants considered that CHC-nurses had become blind to this fact and were no longer able to discern which of the children they meet should be considered overweight.

"“I’ve thought about this regarding BMI. One talks about it a lot nowadays. How good is it actually? I look at the children here, and one sees a lot of quite nice children. Their BMI is high, but when I look at them I can’t see that they are bodily obese. They are big.” (1:13)"

A few kilos of overweight were not regarded with concern, but were rather seen as a family trait. Nevertheless, obesity was regarded by the participants as being a condition among adults and children that today has progressed a long way, and this aroused strong reactions among them.

According to the participants Swedish society has simultaneously realised that there is a concern about overweight and it was the participating CHC-nurses’ conception that, at different levels of society, some attempts have been made to prevent an increase of the problem. Further, the participants believed that the Swedish society has now realised that it would be wrong to allow the problem of overweight and obesity to continue to develop into epidemic proportions as it has in several other countries. Moreover, the CHC-nurses conceived that despite the fact that there exists awareness at the CHC of the concern about the problem, childhood overweight and subsequent obesity is nevertheless a question of priorities and resources.

#### Health seems less important than appearance

Overweight in children was mainly conceived by the CHC-nurses as being steered by how the surrounding world views children. They considered that everyone should, in general be slim today and that the development towards overweight in society tends to include children of an early age. The participants regarded society to be normative, which reflects the parents’ fixation to the official growth charts. CHC-nurses conceived that parents expect their children to look average, like other children and be just about the right size and height.

"“They have to look like the Average-Swede; they have to follow their charts. There’s a lot of focus on this in child health care.” (1:16)"

Nevertheless, overweight in children is about the expectations of others and the fear of alienation. The participants conceived this situation to be the driving force behind parents’ concern about their children’s excess weight. Swedish society does not like overweight in general and it leads to bullying and stigmatisation which, according to the participants, are known to be a common problem among overweight children. It was the CHC-nurses conception that the few parents who actually seek help for their overweight child do so for one or more of three main reasons: worry about their child being bullied for its appearance, worry about their child’s ability to keep up with the other children’s physical pace and worry about their child being alienated. Additionally, the participant CHC-nurses conceived that the parents fear often originates from their own bad experiences related to overweight during their own childhood.

### Overweight in younger children a neglected concern

This category describes the participating CHC-nurses conceptions regarding their own concern about overweight in children at the CHC. Further, it describes their conception of what their work is to be directed towards.

#### Minimisation of the concern for younger children

The participating CHC-nurses conceived overweight in children, primarily obesity in children to be an extensive and serious problem which affects children, families and society. Moreover, they expected the concern to increase with the children’s age. The CHC-nurses considered that overweight is primarily related to hereditary factors and also the fact that overweight in children is not taken seriously during the pre-school period. Most of the participants conceived that it is not before the children turn at least five that CHC-nurses recognise the children’s excessive weight as being a health concern. However, in the participants experience it was not before the children started to approach their first school year that their excess weight began to be a problem for the child and the parents.

"“You cannot help anyone who does not want to be helped. In the beginning the child is protected by the parents. But, the parents will realise the problem sooner rather than later. They will return. If not to the CHC, then they will turn to the school health.” (1:18)"

Very few of the CHC-nurses considered it meaningful to raise the issue with the parents, if the child was 2.5 year or younger. They conceived it important to motivate the family to change the situation for their child, but their main conception was, on the other hand, that it is strictly up to the parents if they want to help reduce their child’s weight or not.

#### Emphasis mainly on diet

The CHC-nurses who participated in this study conceived diet to be of crucial importance for a healthier weight and a healthier child. They considered that they have sufficient knowledge to give general diet counseling but that they lack knowledge beyond the general guidelines aimed to help all those children passing through CHC. Collaboration with the dieticians was considered valuable as the participants conceived that parents of overweight children require more intensified diet counseling than they could offer as nurses. Those nurses who have access to a dietician would rather refer the child to the dietician than give counseling themselves. The participants considered that they had sufficient knowledge regarding the sedentary behavior of children who were overweight however, their conception was that the emphasis in their work was to be directed towards dietary habits.

"“Diet is what we talk most about at CHC. From the first to the last encounter, with every family.” (1:8)"

### Overweight a delicate issue

This category summarises the participating CHC-nurses conceptions concerning the sensitive nature of overweight in children and the difficulties that exist in their work with families with overweight children.

#### Overweight a sensitive subject

The fundamental conception among the participating CHC-nurses was that neither health care personnel or parents nor the Swedish society consider overweight to be desirable. The participants experienced that overweight are something that parents are ashamed of and unwilling to talk about. The participants expressed sympathy for children with overweight and stressed that every child has the same value irrespective of their size and appearance. The CHC-nurses experienced that there was a risk of offending the parents and that there was a need for using neutral terminology and making cautious transcriptions and statements about a child’s weight. If CHC-nurses perceive that the parent is on the defensive or is angry they would rather back away and change the subject. Then the subject isn’t mentioned again until the next encounter - usually a year later. According to the participants, it was regarded to be more legitimate to talk about good eating habits in general without relating to the child’s weight. All of the participants agreed that it was important to safeguard their relations with the parents, and qualities like diplomacy and discretion were the key words used when working with these families.

"“I have learned to be careful with what I say to the parents. It’s difficult to know how to present concern. You don’t want to offend the parent. Already from the start, weight is a bit sensitive for the parents” (1:1)"

#### Overweight in children a provoking concern

The participating nurses related overweight children to meaning more work for the nurses at the CHC. They considered that it required more time and several visits in addition to the general guidelines for children’s care at the CHC. This additional load was something that was considered difficult to keep up with and created further strain on already strained resources. The interviewees stated that there were automatically negative thoughts about the whole family. Some of the participants perceived the parents to be of bad character and to lack good judgment since they gave their children an unhealthy diet**.** The participants also had negative conceptions regarding the family’s everyday life. The CHC-nurses considered the parents to be the cause of their child’s excess weight and meant that the children did not themselves have the ability to alter their situation. The participants found it hard to accept and difficult to talk about the problem of parents allowing their children to become overweight.

"“I feel sorry for the children. It is unnecessary and then I’ll think about what kind of rules exist in the family. One actually gets some negative thought about the whole family/…/ how can they push so much into the child and how do they eat at home? One really wants to help and give good advice and so on.” (1:4)"

The CHC-nurses saw overweight children with growing concern and they understood the problem to be a family matter, where the whole family is in need of support. Further, CHC- nurses also considered that it is important to respect the will of the parents and not to criticise them too much.

### Importance of family lifestyle

This category describes conceptions concerning family lifestyle and what role habits, parents’ socio-economical background, socio-cultural perspectives, parent’s awareness of their child’s excess weight and the parenting role play in childhood overweight.

#### Consequences of parents stressed lifestyle

The CHC-nurses conceived childhood overweight to be a complex problem which is relatively difficult to overcome due to entrenched lifestyle habits among parents. These habits, which primarily involve inadequate diet and lack of exercise, are transferred from the parents to the children in the family. They concluded that full-time working parents are often stressed and lack time to prepare proper meals, which results in several visits per week to hamburger chains and Pizzerias. Further, the participants described that parents are too quick to offer fast food to their children at all times as a compensation for the limited time they spend with them. Sometimes it occurs that mothers use follow-on formula as a compensation for a meal when they think that their child has not been eating enough. The participants also conceived that one parental strategy was to use the television as a babysitter when parents lacked the time to go out and play with their children after work. Overall, the participants expressed that childhood overweight has more to do with the parents than with the child thus a small child is dependent on what the parents give it to eat and what it is occupied with during the day.

"“… an adult causes their own overweight but it is the parents of the small child who teach it the wrong eating habits. The child inherits the parents’ behavior…” (1:3)"

#### Economic conditions and low education level as contributing factors

The participants conceived that the parents’ financial situation is crucial for their choice of diet and activities for their children and they considered it to be common that parents of children with overweight have financial straits and a low level of education. The CHC-nurses concluded that these are the reasons why parents choose cheaper food products with poor nourishment content.

"“…those children who are overweight are those.., how shall I say.., those whose parents have a lower level of education and maybe a poorer economy.., and that I think I can see… The parents just buy the wrong products. They shop and then it just has to be what’s the cheapest, they don’t check the ingredients…” (1:5)"

Furthermore, being in financial straits was regarded by the participants as a reason why so many children do not participate in extra curricula activities. According to the conceptions of the CHC-nurses, poor economy and low education follow hand in hand. The participants considered those parents in financial straits and with a low level of education to be more difficult to reach and stressed that they considered these families to be the ones who should be given priority by the CHC.

#### Preferences differ in socio-culture

A great majority of the CHC-nurses expressed that they conceived that there are cultural differences in what is considered as being the best weight to be. Further, they conceived that in many cultures it is regarded as a sign of health and wealth if children (and even grown-ups in some cases) are plumper. Infants and smaller children were also considered to be stronger and sweeter by their parents if they have more flesh on their bones. Moreover, it was also regarded as a communication problem when parents from certain cultures held a view of a healthy child and a healthy life that does not match the view held by the CHC. The participant’s conception was that some of the foreign mothers were very worried over the fact that their children did not eat enough and it is common that the mothers give both their breast and a supplementary bottle in combination, just to ensure themselves that their child has eaten enough. In addition, the participants considered that some mothers constantly offer their children food and never let them be hungry. Crackers, crisps, sweets, nuts, milk and soft drinks do not count as food, they are snacks, which parents’ do not always understand, in fact, only make a child feel full. The participant’s conception was that adults think that they are being kind when they give treats to children.

"“I don’t actually believe that it has to do with countries but that it actually has to do with knowledge and traditions/…/ And here in Sweden we have, I mean - we have all the resources in the world. You don’t have to fatten the child so it will manage and survive in the case of a possible period of starvation.” (1:15)"

#### Awareness of the problem

The participants expressed that most of the parents of overweight children appear to be aware of their children’s excess weight, but they do not necessarily acknowledge the child’s overweight as a problem. It is rather the parents to those children who follow the official growth chart without deviation, who express concern over their child’s diet and weight. The CHC-nurses stressed that parents of overweight children readily glide away from the fact that their child is heavier than what is recognised as suitable for their age and expect the problem to resolve itself over time.

"“The parents say it is genetic and that he [the child] will thin out when he gets older. They don’t feel any concern at all. My experience is that parents of children with just a little bit extra weight express worry, rather than parents to round children who aren’t a bit concerned.” (1:2)"

Further, the CHC-nurses considered that some parents regard their children as too young for them to need to worry over their excess weight. Nevertheless, the participants still regarded most of the families as being willing to do something about the weight issue. In their encounter with the parents the CHC- nurses experienced that mothers sometimes say what they think the nurses want to hear and embellish reality. Fathers were conceived to tell the truth and to be more honest. Sometimes the participants turned to the children with questions about dietary habits as they expected to get a more honest answer from them.

#### Problem of setting boundaries

The CHC- nurse’s conception was that the role of the parents is important and their ability to set limits for their children appears to be problematic for some of them. Hence, some parents, in families with overweight children, are not clear in their parenthood role and their chosen routines regarding food can be bad or non-existent. The participants concluded that, in some families, there appear to be no rules regarding diet. The nurses expressed that it is difficult for parents to say no to their children since food has an emotional meaning and can be used as a substitute for parental love.

"“…some of the parents give them [their children] food as soon as they are screaming. It´s a way to calm them down.” (1:12)"

Another aspect experienced by the participant nurses was that parents do not always understand that children have small stomachs and therefore they expect their children to eat unreasonably large amounts of food. The participants concluded that there is not always an understanding about the relationship between children’s need of nutrition and children’s development and growth. The CHC-nurses conceived this to be the reason that children who fuss with food were considered to be a stress factor for some mother’s, for example, those who lack the pleasurable experience of sitting down to eat together with their children.

## Discussion

In accordance with the aim of this study, we found a wide variation in CHC-nurses conceptions of overweight in children. Several descriptions emerged regarding overweight children, all of which were regarded as difficult to deal with at the CHC due to some of their core elements. The findings of the present study have, to some extent, features that are parallel to previous findings regarding the attitudes and beliefs among health care personnel and parents regarding overweight children [15,24,25,31-48]. These findings will be summed up and dealt with further in this text following the order of the presented results.

This study adds to the knowledge related to overweight and obese children and the acceptance by society of the problems of children’s excess weight. This acceptance affects CHC-nurses conceptions of what is a healthy weight for a child, which is an important finding that adds to the existing knowledge. Nurses consider that the view of both the Swedish society and the health care professionals about the healthy weight of preschool children has changed. Earlier views have been revised due to the increased proportion of overweight children in society. Nevertheless, the CHC-nurses in this study emphasised the seriousness of childhood overweight and their experience was that it increases with a child’s age.

There is a vast difference between being aware of one’s child excess weight and to actually realise that it is overweight. The participating CHC-nurses expressed that most of the parents are aware of their children’s overweight, but the few parents who seek help for their overweight child do not express their concern about the health issues. Hence, the participating CHC-nurses emphasised that it is the child’s appearance and the parent’s fear of their child being bullied that leads them to seek help from the CHC. This finding is supported by research indicating that mothers´ focus more on concerns over stigmatisation and bullying and less on the physical ramifications of the problem [46,47].

When asked, more than 90 percent of the public believed that it is the parents who have the main responsibility for reducing childhood overweight [35]. This result is in line with our finding regarding CHC-nurse’s perception that it is up to the parents whether or not they choose to make a change in their children’s weight. Paradoxically, all of the CHC-nurses in our study perceive childhood overweight as a problem of great concern and emphasise that it is important to motivate the family to change and not just to criticise the parents. At the same time, a great majority of them did not take overweight among children in their early preschool years seriously. The CHC´s responsibility for a child extends until the child’s care is transferred to the school health care system at 6 years of age. It may be possible that this fact makes it easier for the CHC-nurses to put the problem aside as at six years they are able to hand the responsibility for the child and its overweight problem over to the school nurses. However, to the best of our knowledge, there are no other studies implying such findings.

The participating CHC-nurses experience having negative thoughts towards parents and blame them for their children’s excess weight. These negative thoughts are expressed in terms of being a burden for the CHC as these families are time consuming. One reason for these experiences may be that CHC-nurses are uncomfortable with their encounters with parents who are often overweight themselves [25]. Moreover, the CHC-nurses expressed that lack of time, financial resources and staff, constitute barriers against working with childhood overweight, which is supported by Moyers et al. [15]. Furthermore, some of the CHC-nurses in our study expressed that fathers and children were more likely to tell the truth about the children’s dietary habits than the mothers. As far as we know, there are no studies dealing with the gender differences between parents concerning honesty in reporting family dietary habits. Traits like negative thoughts, suspicion and blame among health care personnel are reflected in their encounter with the parents of overweight children. Perceptions of help- seeking experiences among parents of overweight children indicate that they occasionally experience the health care professional’s response as negative and dismissive [31]. Our present knowledge about parents of overweight children facing stigmatisation is based upon literature from the fields of both nursing and medicine dealing with difficult patients and parents. These parents are often referred to in terms of blame and stigmatisation [32-34]. There is therefore an imminent risk that negative thoughts and lack of resources of CHC-nurses are reflected as negative attitudes in an encounter with an overweight child and its parents, which in turn may affect the parent-nurse relationship.

The nurses who participated in this study indicated that some parents have difficulties in setting limits for their children’s demands regarding food preferences, which is supported by previous research [24,36]. Our findings show that the CHC- nurses conceived that parental stress and lack of time was compensated by fast food and that it was difficult for parents to say no to their children since food has an emotional meaning and could be used as a substitution for parental love. Food can be used as a tool for coping with stress and as a tool in parenting [24,37], which is in line with our results.

One of the strongest social determinants of childhood overweight and obesity is known to be the education of mothers [40]. The CHC-nurses in this study stressed that it was important to give priority to families with a poor economy and a low level of education, as children in these families are especially vulnerable regarding childhood overweight. Our findings are supported by a previous qualitative study [41], which showed that parents from high and low socio-economic groups differ in their ways of handling overweight related health problems and therefore intervention strategies should be individually tailored to each group and family. Further, there is a body of literature showing that mothers with a lower educational level are less likely to recognise their children as being overweight, despite their children’s obvious excess weight [38,42,43]. Additionally, children’s probability of becoming overweight is linked to their mother’s time and hours spent working per week [38]. Our study show that parental lifestyles and time spent with children is perceived to have a great impact on dietary intake and thereby children’s weight, which is also supported by Jackson et al. [39].

A study by Heubeck [44] indicates that it is least likely that poorer and immigrant subgroups identify childhood overweight as a serious problem. The CHC-nurses in our study stressed that existing cultural differences regarding eating habits and body perception, affect the probability for childhood overweight in families from different cultures. In their study, Fisher and Birch [45] reported that it was more important to parents belonging to lower socio- economic groups that there was food available and that their children consumed a sufficient quantity of food. There is an equivalent finding in this present study regarding mothers constantly offering their children food and never letting them feel hungry.

The overall conception of the participating CHC-nurses was that overweight in children becomes a concern first when the children reach school-age. The participants considered it important to motivate the family to change their habits, but it is up to the parents whether or not they make dietary changes that will bring their child’s weight into line. The CHC is voluntary and this situation may result in the CHC-nurses making less effort and reducing their persistence to raise the subject with the parents. All of what is mentioned above, as well as the fact that overweight in children is perceived to be a sensitive subject may lead to the risk of tactical underestimation of a child’s overweight by the CHC and thereby lead to insufficient treatment [48].

### Methodological considerations

There are certain strengths, as well as limitations, to consider that may affect the interpretation of the present study. Strengths are that all of the interviews and the analysis were conducted by the first author (GEI) and all of the participants were asked the same initial question. Almost all of the CHC-nurses (n = 19) who were requested to participate in this study wished to be included apart from one. This meant that 18 CHC-nurses represented the 17 different CHCs included in the study. Further strengths are that the interviews were experienced as positive by the participants who were generally open and easy to talk to. There existed an understanding of the profession-specific terms and routines used in CHC due to the professional background of the first author (GEI), which could possibly have facilitated the interview process.

The CHCs included were situated in both well to do and poor areas of the four municipalities chosen and the CHCs differed in both the composition of RSCNs´ and DNs´ and the number of employees per unit. They all had the same local director but different responsible clinic managers. This should not have influenced the findings as they all have the same general guidelines for CHC. The limitation of this study is that the findings can only be valid for those nurses who participated in the present study, which means that a gender perspective is missing. The number of eligible male RSCNs and DNs in CHC is low in the southern part of Sweden, however, all those eligible took part in this study. Further, the authors believe that the result of this study might be transferable to similar contexts of CHC as the sample of CHCs represented both urban and rural areas.

Another strength is that the findings from the study were continuously discussed back and forth between the authors during the analysis. Further, this interdisciplinary cooperation facilitated the guarding against the effects of personal biases and thereby improves the trustworthiness. The derived conceptions and later the categories of description were regularly scrutinised by the third author (GA), who is experienced in phenomenographic analysis, to attain greater rigour.

This area of research is still unexplored and there are few studies conducted involving CHC-nurses. This study adds to the accumulated knowledge the perceptions of the CHC-nurses, who apart from other health care personnel are those who most frequently encounter preschool children with overweight and obesity at the CHCs.

## Conclusion

The CHC- nurses’ conceptions regarding overweight children provide a comprehensive and complex picture of the issue. According to the participating CHC-nurses, today’s society also generally accepts childhood overweight. The participating nurses’ conceptions also elucidate the fact that overweight children are now so common they no longer stand out, which has affected the view of parents and nurses regarding what is a healthy weight for a preschool child.

The participating CHC-nurses’ experienced that it was difficult to deal with childhood overweight at the CHC because parents of overweight children often failed to acknowledge their children’s overweight as a problem. CHC-nurses experienced difficulties in raising the subject of childhood overweight with these parents. Some of the CHC-nurses in this study experienced ambivalent feelings regarding childhood overweight and obesity and there was a tendency by CHC-nurses to minimise the concern about overweight in younger children.

## Implications for practice

The findings from this study elucidate the need for continuing education about childhood overweight and weight assessment with emphasis on pre-school children in CHC. Raising the awareness of CHC-nurse’s ambivalent feelings about childhood overweight is important since it may affect the parent-nurse relationship and thereby the parent’s and the nurse’s effort to influence the children’s weight. Further, the findings from this study may be of value for other health care personnel and professionals working with these families, since awareness of existing perceptions may facilitate in their own work with these families. The authors suggest that CHC-nurses should work with person centered counseling and towards empowering the families, as recommended by the existing CHC guidelines.

## Competing interests

The authors declare that there are no competing interests.

## Authors’ contributions

GEI and AKD participated in the design of the study and developed the interview guide. GEI conducted all of the interviews, made the initial analysis of the interview transcripts and drafted the manuscript. AKD and ACB assisted in the initial analysis of the interview transcripts. All of the authors discussed the analysis amongst them and regularly made comments on the reporting and the draft of the manuscript. All of the authors read and approved the final manuscript.

## Pre-publication history

The pre-publication history for this paper can be accessed here:

http://www.biomedcentral.com/1471-2296/13/57/prepub
